# Improvement in Chimneys

**Published:** 1883-01

**Authors:** 


### IMPROVEMENT IN CHIMNEYS.

The best chimneys are made by inclosing hard baked glazed pipe in a thin wall of bricks. Such chimneys will not only draw better than those made in the usual way, but there will be less danger from " defective flues." A four-inch wall of bricks between us and destruction by fire is a frail barrier, especially if the work is carelessly done or the mortar has crumbled from the joints. To build the chimneys with double or eight-inch walls makes them very large, more expensive, and still not as good as when they contain the smooth round flues. To leave an air chamber between them for ventilating, is better than to open directly into the smoke flue, because it will not impair the draught for the fire, and there will be no danger of a sooty odor in the room when the circulation happens to be downward, as it will be occasionally. The outside chimney if there is one, should have an extra air chamber between the very outer wall and the back of the fireplace to save heat, a precaution that removes to a great extent the common objection to such chimneys. A very large per cent. of fires come from defective chimneys.

